# COVID‐19: what is the disaster?

**DOI:** 10.1111/1469-8676.12890

**Published:** 2020-05-20

**Authors:** Ilan Kelman

**Affiliations:** ^1^ Institute for Global Health and Institute for Risk and Disaster Reduction University College London London WC1E 6BT UK

The COVID‐19 pandemic conforms to key baseline conclusions which have emerged from disaster anthropology over past decades. First, that natural disasters rarely exist, because disasters are social, arising from a combination of hazard and vulnerability, with vulnerability as the causative factor. Second, that the disaster occurs at multiple levels simultaneously, with responses to a hazard exposing as many vulnerability problems as the original hazard.

Regarding the misnomer ‘natural disaster’, the hazard here is the new coronavirus which could have been dealt with before it became an epidemic or a pandemic. At its origin in Wuhan, China, doctors swiftly identified the emergence of a new disease, reported their concerns about the dangers and worked out biological aspects of the virus. The response from the authorities included intimidation and silencing of the medical professionals, seeking to cover up the possibility of an outbreak.

Once the pandemic took hold, the failings of health systems around the world became evident. Especially in wealthier countries, many governments had long had pandemic plans indicating the need for more robust health systems, from improved disease surveillance to paying medical personnel appropriately and to having protective equipment available. The failure to heed these warnings, alongside the lack of healthcare accessible to everyone in the USA, meant that the hazard could not be addressed effectively and vulnerability fundamentals were revealed.

Also on the vulnerability side, deep questions need to be explored covering why humanity disturbs ecosystems to the point that microbes jump species, creating new hazards – as happened with HIV and Ebola in addition to the new coronavirus – and why food markets operate without proper oversight or hygiene. From both hazard and vulnerability perspectives, the pandemic disaster was not natural, but was entirely socially caused.

The pandemic was not the only disaster. Without disputing the need for the lockdowns seen around the world, this approach’s consequences represent further layers of the COVID‐19 disaster. Expectations of further disaster layers incorporate more mental health issues, medical problems from augmented stress and worsened diet, self‐harm including suicide attempts, domestic violence and substance use. All these are poorly treated epidemics across societies already, but were rarely considered fully within the context of ordering lockdown.

The destruction of a lifetime’s dedication to building up a small business (closed during lockdown) and not holding family members’ hands as they die add to physical and mental health impacts. It is telling that the phrase ‘social distancing’ was used first, only to be replaced by ‘physical distancing’ on the important premise that we need to remain as socially close as possible without physical proximity.

The lockdowns nonetheless saved, at minimum, tens of thousands of lives. Part of pandemic planning and dealing with a pandemic disaster is to incorporate immediately the disastrous aspects brought by lockdowns. None of this knowledge is new. It was all available long before the virus appeared at the end of 2019, yet once again we witness the failure to use what we know to prevent disasters.

